# Dysphagia and aspiration during a Parkinson's hospitalization: a care partner's perspective and recommendations for improving standards of care

**DOI:** 10.3389/fnagi.2023.1258979

**Published:** 2023-10-09

**Authors:** Annie Brooks

**Affiliations:** Strategic Initiatives, Parkinson's Foundation, New York, NY, United States

**Keywords:** Parkinson's disease, hospitalization, dysphagia, aspiration pneumonia, caregiving

## Abstract

People with Parkinson's disease have a significantly increased incidence and risk of aspiration pneumonia when compared to those without. Aspiration pneumonia associated with dysphagia (swallowing issues), which is the leading cause of death among people with Parkinson's disease, accounting for 25% of Parkinson's deaths. There is relatively limited evidence of the most effective strategies to balance the competing needs of each Parkinson's patient as providers aim to prevent, diagnose, and manage dysphagia. Exacerbated, and in part caused, by the intricacies of dysphagia and Parkinson's disease, there is still limited understanding among hospital providers and the Parkinson's community regarding the most appropriate measures to prevent and manage dysphagia in Parkinson's disease. The Parkinson's Foundation Hospital Care Recommendations identified the prevention and management of dysphagia as a care standard necessary to eliminate harm and attain higher reliability in care. This article discusses key components of dysphagia management in the hospital, provides a case example to demonstrate the challenges that people with PD and their care partners experience in the hospital related to dysphagia, and offers recommendations on how to better manage dysphagia and involve care partners in PD hospital care.

## Introduction

Of the nearly one million people living with Parkinson's disease (PD) in the U.S., an estimated one in six will experience avoidable complications in the hospital often related to medication management, mobility, and dysphagia (Aminoff et al., 2011; Hassan et al., 2013; Zeldenrust et al., 2020). Dysphagia, or impaired swallowing, is a common PD symptom that can lead to serious problems for people with PD in the hospital, including aspiration pneumonia, increased mortality, and longer hospitals stays (Di Luca et al., 2021; Gong et al., 2022). PD medication adjustments that may seem insignificant can lead directly to dysphagia and indirectly to aspiration pneumonia (Lenka et al., 2020). The use of antipsychotic medications is also associated with an increased risk of aspiration pneumonia in older PD patients (Huang et al., 2021). The Parkinson's Foundation Hospital Care Recommendations, which outline optimal hospital care for people with PD, identify dysphagia management as one of the five key standards of quality hospital care (Parkinson's Foundation, 2023).

Bringing a care partner to the hospital who can advocate for one's needs is critical for people with PD because staff may not fully understand PD or know how to properly manage a patient's PD symptoms, including those related to swallowing. This article discusses key components of dysphagia management in the hospital, provides a case example to demonstrate the challenges that people with PD and their care partners experience in the hospital related to dysphagia, and offers recommendations on how to better manage dysphagia and involve care partners in PD hospital care.

## Dysphagia management in the hospital

### Screening and assessment

Diagnosing dysphagia typically involves three steps—a screening test, a clinical assessment, and an instrumental assessment (Riera et al., 2021). However, the diagnostic approach and the staff involved often vary in the inpatient setting because there is no nationally recognized standard protocol.

Dysphagia is symptomatically multidimensional, and therefore, a multidisciplinary approach to assessment is recommended (Cordier et al., 2023). While speech language pathologists (SLPs) are qualified to lead the assessment, diagnosis, and management of dysphagia (American Speech-Language-Hearing Association, 2016), limitations of SLP staffing and availability make this difficult. As an alternative, initial dysphagia screening tests may be conducted by nurses, with a referral to SLP for further assessment if necessary (Fedder, 2017).

Screening tools that provide optimal diagnostic performance in patients with PD are essential regardless of whether they are being completed by SLPs or nurses. Despite this, two of the most common dysphagia screening tools in use today across settings and patient populations, namely, volume-dependent water swallow tests and subjective patient reports, are both inadequate for patients with PD (Speyer et al., 2021; Dumican et al., 2023). Although there is not yet evidence to support a strong screening tool (Frank et al., 2020), several validated assessment tools do exist (Tomita et al., 2018; Christmas and Rogus-Pulia, 2019).

### Care partner advocacy

Parkinson's care partners play an important role during hospital stays. They can assist by providing staff with educational materials, accurately explaining the patient's medication schedule, and conveying the patient's wishes. Having a care partner present can be helpful to all patients as the impact of one's disease can make it more difficult to express one's needs and understand information shared by one's healthcare team (Fenton et al., 2022). Studies have found that the presence of family members during hospitalization increases patient comfort and can even help identify errors that may otherwise be missed (Correia et al., 2020).

The advocate role is also important to many care partners. One study highlighted that nearly 50% of care partners wanted to influence the care provided to their loved ones in the hospital (Lindhardt et al., 2008) as they hold essential information about their loved one's condition, routine, and overall needs. Defining optimal care for each patient often requires direct dialogue with care partners (Bragstad et al., 2014). When healthcare staff work together with patients and families, “the quality of healthcare increases, costs decrease, and patient satisfaction improves (Smith et al., 2022).”

However, care partners are frequently not kept informed while their loved one is hospitalized (Fields et al., 2023) as some providers view family involvement as risky or time consuming (Smith et al., 2022). Families often recognize this hesitance to trust care partner input, which decreases their confidence in staff and satisfaction of care (Whittamore et al., 2014). Among many care partners, there is a sense that hospital policies may contribute to insufficient communication because there are limited established protocols to ensure care partners' access to information (Giguere et al., 2018). When care partners are not kept informed about their loved one's care, they struggle to influence the decisions being made or help improve the care being delivered (Smith et al., 2022).

## Case example

This case example is provided to illustrate the dysphagia-related challenges people with Parkinson's disease and their care partners face while in the hospital as well as the challenges care partners face when attempting to advocate for their loved ones. The interviewee, a Parkinson's Foundation volunteer and care partner to her husband with PD, agreed to participate in a virtual semi-structured interview with the author. The goal of the interview was to describe her husband's experience as a person with Parkinson's disease in the hospital and the interviewee's experience as his care partner.

### Case summary

**Figure 1:**
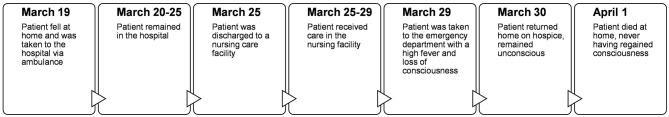
Timeline.

A 74-year-old man with PD diagnosed at 60 years and now with known dementia and dysphagia was admitted to a rural critical access hospital after a fall. The patient was admitted and hospitalized for 5 days for assessment before being discharged to a large continuing care retirement community to receive skilled care and intensive therapy. Four days later, after developing a fever and becoming unresponsive, he was readmitted to the hospital and was subsequently diagnosed with aspiration pneumonia. The following day he was transitioned to home hospice where he died 2 days later (Figure 1).

### Care partner perspective

On 19 March 2021, the patient was taken to the hospital *via* ambulance after falling, unable to get up, seeming disoriented and in significant pain. The care partner (CP) accompanied him to the hospital where he was admitted. Due to COVID-19 protocols, she was only permitted to be with him for 6 h per day. This was especially worrisome for the CP because her husband's dementia had progressed to the point that he could not communicate his needs.

CP attempted to advocate for her husband's Parkinson's-specific needs throughout his stay speaking to numerous hospitalists, nurses, and nurse aids. Upon arrival, she explained to the hospitalist that her husband had trouble swallowing and that she typically gave him his medication with either applesauce or yogurt, but medications were withheld rather than given with thickened liquid. As an education and advocacy tool, CP brought and used her hospital safety kit, provided by the Parkinson's Foundation. Included in the kit are tips to share if swallowing is an issue, including “sit up while eating, tuck in your chin each time you swallow,” (Parkinson's Foundation, 2022) but she did not see any aspiration prevention measures implemented and received no information about the offsite SLP being contacted.

CP identified nurse aids as particularly crucial team members, given the amount of time they spent with her husband, but felt they were the least receptive to input about her husband's care needs and lacked a sufficient understanding of his risk of aspiration. She viewed her husband's care in the hospital as problematic, acknowledging the impact the pandemic had on her ability to advocate and on the healthcare facilities' capacity to provide quality care. She also felt the lack of staff knowledge about risk factors of dysphagia and aspiration pneumonia was the primary factor leading to his steep decline. She highlighted the key role of the CP in PD management, sharing her frustration that she was unable to be more present as an advocate and that hospital staff were unreceptive to her input, particularly related to his swallowing.

### Case example limitations

A key limitation of the case example is the presentation of a single perspective without access to the medical chart or input from medical care providers. Additionally, the impact of the visitation limitations implemented during the height of the COVID-19 pandemic must be considered.

Establishing meaningful channels of communication became especially challenging in the throes of COVID-19 when the physical presence of care partners was discouraged and often significantly limited (Bragstad et al., 2014). An integrative review of 17 articles published in 2021 focused on the impact of COVID-19 visitation limitations identified numerous unintentional consequences of the absence of care partners, including significant decline in health and wellbeing of family members, adverse health effects, and decreased quality of life (Hugelius et al., 2021).

## Discussion

As we consider the next steps in improving dysphagia management and aspiration pneumonia prevention among people with Parkinson's disease in the hospital, evidence-based practices for clinicians must be established, ideally as part of nationally recognized standards of care. We also must ensure that the Parkinson's communities—care partners and people with PD—are informed collaborators throughout hospital care. In the next section, we will discuss both.

### Recommendations for dysphagia best practices in the hospital

Hospitals need clear best practices for assessing and treating dysphagia in patients with PD. Dysphagia prevention and management is one of the five care standards outlined in the Parkinson's Foundation Hospital Care Recommendations. The standard states that “All people with Parkinson's should undergo screening for dysphagia within 24 h, with measures taken to minimize the risk of aspiration pneumonia, as needed (Parkinson's Foundation, 2023).”

These recommendations represent a step forward in establishing best practices but do not define the standard protocol for institutions to utilize. With consistent protocols, sufficient implementation will be uneven across institutions, and patients with PD will remain at risk. More rigorous research on how to best assess, prevent, and treat dysphagia is needed.

#### Requirement 1: standard screening of swallowing abilities

To reach consensus on a standard screening protocol, additional research must validate effective screening tool(s) for identifying dysphagia in PD patients, ensuring the feasibility of use across hospital settings. Without appropriate screening tools, hospitals will need support in prioritizing the timely use of validated assessment tools.

#### Requirement 2: standard protocol for minimizing aspiration pneumonia risk

Generally, it is considered appropriate but is a rarely established policy for people with Parkinson's disease in the hospital to eat only while maintaining an upright posture. Establishing this as a policy would still require the navigation of administrative processes in individual hospitals.

Standardizing hands-on practices, such as supervising meals, may be more challenging. More research is needed to demonstrate when this level of care is necessary.

#### Requirement 3: standard protocol for medication management when dysphagia is identified

Since PD medications contribute to the maintenance of the ability to swallow, a customized approach to medication management in PD patients with mild-to-moderate dysphagia may prevent the continued deterioration of swallowing function. Clinical tools that can clarify the severity of dysphagia could make this possible. Identification or development of these tools is necessary before we can recommend appropriate medication protocols based on the severity.

Broadscale adoption of standards will require a national dissemination and education plan, buy-in from health systems and quality improvement organizations, and additional research into the areas of limited evidence.

### The role of patients and care partners

In addition to the need for reliable tools and evidence-based best practices in PD care, there is also a need to educate the Parkinson's community, and in particular, care partners of people with Parkinson's disease, about what they can do to help prevent aspiration pneumonia in the hospital.

Care partners are essential as advocates during hospitalization and should be acknowledged as an important member of the care team. Without staff respect for care partner input, however, the success of advocacy is limited. For example, in the case example, even though the care partner came equipped with tools in the Hospital Safety Kit, she struggled to impact care. It is important for hospitals to adopt standard protocols that honor the care partner role and create opportunities for their input on the care being provided.

Future research can strengthen care partner advocacy efforts by offering evidence-based answers to complex questions about how care partners can best monitor and advocate for their loved ones needs related to medication management, swallowing, dysphagia, and aspiration pneumonia.

## Conclusion

The Parkinson's Foundation, in collaboration with key clinical and community stakeholders, is committed to understanding the challenges associated with dysphagia in the hospital and identifying strategies and best practices for improving care and outcomes. We prioritize the inclusion of care partners and people with PD in our process and invite all healthcare systems and leadership to join us as we work together to eliminate preventable harm in the hospital for people with Parkinson's disease.

## Data availability statement

The original contributions presented in the study are included in the article/supplementary material, further inquiries can be directed to the corresponding author.

## Author contributions

AB: Writing—original draft.

## References

[B1] American Speech-Language-Hearing Association (2016). Scope of Practice in Speech-Language Pathology [Scope of Practice]. Available online at: www.asha.org/policy/ (accessed January 06, 2023).

[B2] Aminoff M. J. Christine C. W. Friedman J. H. Chou K. L. Lyons K. E. Pahwa R.. (2011). Management of the hospitalized patient with Parkinson's disease: current state of the field and need for guidelines. Parkinson. Relat. Disord. 17, 139–145. 10.1016/j.parkreldis.2010.11.009 21159538 PMC3070297

[B3] Bragstad L. K. Kirkevold M. Foss C. (2014). The indispensable intermediaries: a qualitative study of informal caregivers' struggle to achieve influence at and after hospital discharge. BMC Health Serv. Res. 14, 331. 10.1186/1472-6963-14-331 25078610 PMC4119054

[B4] Christmas C. Rogus-Pulia N. (2019). Swallowing disorders in the older population. J. Am. Geriat. Soc. 67, 2643–2649. 10.1111/jgs.16137 31430395 PMC7102894

[B5] Cordier R. Speyer R. Martinez M. Parsons L. (2023). Reliability and validity of non-instrumental clinical assessments for adults with oropharyngeal dysphagia: a systematic review. J. Clin. Med. 12, 721. 10.3390/jcm12020721 36675650 PMC9861493

[B6] Correia T. S. P. Martins M. M. F. P. da S. Barroso F. F. M. (2020). The family and safety of the hospitalized patient: an integrative literature review. Portuguese J. Public Health 38, 129–140. 10.1159/000511855

[B7] Di Luca D. G. McArthur E. W. Willis A. Martino R. Marras C. (2021). Clinical and economic outcomes associated with dysphagia in hospitalized patients with Parkinson's disease. J. Parkinson's Dis. 11, 1965–1971. 10.3233/JPD-212798 34366378

[B8] Dumican M. Thijs Z. Harper K. (2023). Clinical practice patterns of speech-language pathologists for screening and identifying dysphagia. Int. J. Lang. Commun. Disord. 1–15. 10.1111/1460-6984.12921 37376825

[B9] Fedder W. N. (2017). Review of evidenced-based nursing protocols for dysphagia assessment. Stroke. 48, e99–e101. 10.1161/STROKEAHA.116.011738 28280136

[B10] Fenton A. Stevens S. Cost Z. Bickford J. Kohut M. Jacobs E. A.. (2022). Patients' and caregivers' experiences of hospitalization under Covid-19 visitation restrictions. J. Hosp. Med. 17, 819–826. 10.1002/jhm.12924 35920080 PMC9538139

[B11] Fields B. Werner N. Shah M. N. Hetzel S. Golden B. P. Gilmore-Bykovskyi A.. (2023). Adapting and testing the care partner hospital assessment tool for use in dementia care: protocol for a 2 sequential phase study. JMIR Res. Prot. 12, e46808. 10.2196/46808 37347517 PMC10337334

[B12] Frank U. Radtke J. Nienstedt J. C. Pötter-Nerger M. Schönwald B. Buhmann C.. (2020). Dysphagia screening in Parkinson's disease. A diagnostic accuracy cross-sectional study investigating the applicability of the gugging swallowing screen (guss). Neurogastroenterol. Motil. 33, e14034. 10.1111/nmo.14034 33217102

[B13] Giguere A. M. Farmanova E. Holroyd-Leduc J. M. Straus S. E. Urquhart R. Carnovale V.. (2018). Key stakeholders' views on the quality of care and services available to frail seniors in Canada. BMC Geriat. 18, 290. 10.1186/s12877-018-0969-y 30477438 PMC6260583

[B14] Gong S. Gao Y. Liu J. Li J. Tang X. Ran Q.. (2022). The prevalence and associated factors of dysphagia in Parkinson's disease: a systematic review and meta-analysis. Front. Neurol. 13, 1000527. 10.3389/fneur.2022.1000527 36277913 PMC9582284

[B15] Hassan A. Wu S. S. Schmidt P. Dai Y. Simuni T. Giladi N.. (2013). High rates and the risk factors for emergency room visits and hospitalization in Parkinson's disease. Parkinson. Relat. Disord. 19, 949–954. 10.1016/j.parkreldis.2013.06.006 23835430

[B16] Huang K.-H. Kuo W.-Y. Kuan Y.-H. Chang Y.-C. Tsai T.-H. Lee C.-Y. (2021). Risk of pneumonia is associated with antipsychotic drug use among older patients with Parkinson's disease: a case-control study. Int. J. Med. Sci. 18, 3565–3573. 10.7150/ijms.63246 34522183 PMC8436093

[B17] Hugelius K. Harada N. Marutani M. (2021). Consequences of visiting restrictions during the Covid-19 pandemic: an integrative review. Int. J. Nurs. Stud. 121, 104000. 10.1016/j.ijnurstu.2021.104000 34242976 PMC8196532

[B18] Lenka A. Mittal S. O. Lamotte G. Pagan F. L. (2020). A pragmatic approach to the perioperative management of Parkinson's disease. Can. J. Neurol. Sci. 48, 299–307. 10.1017/cjn.2020.211 32959743

[B19] Lindhardt T. Nyberg P. Hallberg I. R. (2008). Collaboration between relatives of elderly patients and nurses and its relation to satisfaction with the hospital care trajectory. Scandinavian J. Caring Sci. 22, 507–519. 10.1111/j.1471-6712.2007.00558.x 19068046

[B20] Parkinson's Foundation (2022). Aware in Care Hospital Action Plan. Available online at: https://www.parkinson.org/sites/default/files/documents/hospital-action-plan.pdf (accessed January 06, 2023).

[B21] Parkinson's Foundation (2023). Parkinson's Foundation Hospital Care Recommendations. Available online at: https://www.parkinson.org/sites/default/files/documents/hospital-care-recommendations-april2023.pdf (accessed January 06, 2023).

[B22] Riera S. A. Marin S. Serra-Prat M. Tomsen N. Arreola V. Ortega O.. (2021). A systematic and a scoping review on the psychometrics and clinical utility of the volume-viscosity swallow test (V-VST) in the clinical screening and assessment of oropharyngeal dysphagia. Foods 10, 1900. 10.3390/foods10081900 34441677 PMC8391460

[B23] Smith H. Grindey C. Hague I. Newbould L. Brown L. Clegg A.. (2022). Reducing delayed transfer of care in older people: a qualitative study of barriers and facilitators to Shorter Hospital stays. Health Expect. 25, 2628–2644. 10.1111/hex.13588 36193616 PMC9700150

[B24] Speyer R. Cordier R. Farneti D. Nascimento W. Pilz W. Verin E.. (2021). White Paper by the European society for swallowing disorders: screening and non-instrumental assessment for dysphagia in adults. Dysphagia 37, 333–349. 10.1007/s00455-021-10283-7 33787994 PMC8009935

[B25] Tomita S. Oeda T. Umemura A. Kohsaka M. Park K. Yamamoto K.. (2018). Video-fluoroscopic swallowing study scale for predicting aspiration pneumonia in Parkinson's disease. PLoS ONE 13, e0197608. 10.1371/journal.pone.0197608 29874285 PMC5991364

[B26] Whittamore K. H. Goldberg S. E. Bradshaw L. E. Harwood R. H. (2014). Factors associated with family caregiver dissatisfaction with acute hospital care of older cognitively impaired relatives. J. Am. Geriat. Soc. 62, 2252–2260. 10.1111/jgs.13147 25516022

[B27] Zeldenrust F. Lidstone S. Wu S. Okun M. S. Cubillos F. Beck J.. (2020). Variations in hospitalization rates across Parkinson's Foundation Centers of Excellence. Parkinson. Relat. Disord. 81, 123–128. 10.1016/j.parkreldis.2020.09.006 33120073

